# Proximal Trauma Increases Risk of Venous Thrombosis in Soft Tissue Reconstruction of Open Lower Limb Fractures

**DOI:** 10.3389/fsurg.2020.574498

**Published:** 2021-01-15

**Authors:** Nilay G. Yalcin, Frank Bruscino-Raiola, Scott Ferris

**Affiliations:** Plastic, Hand and Faciomaxillary Surgery Unit, Alfred Health, Melbourne, VIC, Australia

**Keywords:** limb salvage, microsurgery, trauma, complication, thrombosis

## Abstract

Lower limb salvage after major trauma is a complex undertaking. For patients who have suffered multi-level trauma to their lower limb we postulated that pelvic injury or ipsilateral lower limb injury proximal to the site of a free flap may increase the rate of post-operative complications. All patients who underwent lower limb free flap reconstruction as a result of acute trauma between January 2010 and December 2017 were included. The patients were divided into the study group (50 patients), who sustained a lower limb or pelvic injury proximal to the free flap site, and control group (91 patients) who did not sustain proximal lower limb or pelvic trauma. Complication rates were compared between the two groups. Overall, the proximal trauma group anastomotic thrombosis rate of 18.0% was significantly higher than the control group thrombosis rate of 2.2%. There was no statically significant difference in rates of hematoma, swelling or infection. Flap loss rate in the proximal trauma group was 4.0%, compared to the control group at 2.2%. All patients with a failed flap went onto have a successful reconstruction with a subsequent flap in the acute admission and there were no amputations. In the proximal injury study group despite the significantly increased rate of microvascular thrombosis requiring revision, the ultimate primary free flap survival rate was still 96%. Overall, severe coexisting proximal trauma predicted a higher venous microvascular complication rate but was not a contraindication to limb salvage.

## Introduction

Traumatic lower limb defects require a multidisciplinary approach for the salvage of the limb. The decision to reconstruct the limb is to allow the patient to ultimately restore physical, social and economic well-being (1–4). The primary aim of flap reconstruction is to achieve adequate soft tissue coverage of underlying structures to promote bony healing, preserve tendinous function, protect from infection and act as a vehicle for the transport of antimicrobial agents to the site of infection, thereby optimizing healing and function. For severe lower limb trauma, the basic principles include aggressive, often multiple debridements (5–8), skeletal stabilization and early soft tissue coverage (9–11). Where there is significant or extensive trauma to local tissue, particularly in the distal third of the leg or foot, the best alternative for wound coverage is usually a free flap (12–14). Fasciocutaneous, myocutaneous or muscle-only free flaps are often utilized, however the choice of tissue transfer may also include vascularized bone in order to bridge a segmental bone loss (15, 16).

If the patient is not deemed suitable for reconstruction or reconstruction fails, the alternative treatment is amputation. The success rate of free flaps depends on a number of factors. Age, infection, delayed coverage and presence of co-morbidities have been associated with a higher risk of flap failure in previous studies (17–20). It is generally agreed that early reconstruction is associated with a lower incidence of infection. However, the definition of “early” varies between different studies, extending between 1 and 15 days (5–7, 9, 20–27).

It is usual in major trauma for patients to sustain injuries at multiple sites. Few studies however, have evaluated the success rate of flap surgery in the setting of associated injuries. A previous study by Rinker et al. focusing on a pediatric population did not establish any link between mechanism or type of injury with complication rate in 26 patients in total (28).

The aim for this study was to evaluate the outcomes of adult lower limb free flaps in traumatic lower limb wounds when there was a concomitant pelvic or ipsilateral lower limb injury proximal to the site of the free flap, which we hypothesized could affect free flap complication rates and patient outcomes.

## Materials and Methods

All patients undergoing lower limb free flap reconstruction as a result of acute leg and/or foot open fractures and/or dislocations between January 2010 to December 2017 were included in this study. The patients were identified and data collected from our lower limb trauma database and medical records. Data collected included patient co-morbidities, smoking status, age, type of flap, type of arterial and venous anastomoses and complications.

All eligible patients were divided into two groups- one with no injury of the lower limb/pelvis proximal to the free flap site (control group) and the second group with concomitant significant injury to the lower limb/pelvis proximal to the flap site (proximal trauma group). The nature of proximal injuries included, but was not limited to pelvic, femoral or knee fractures or dislocations. One patient from the proximal trauma group sustained a degloving injury over a femoral fracture but otherwise the remaining proximal injuries were all closed fractures or dislocations. There was no recorded direct vascular injury in any patient within the study group. The outcomes between the two groups were compared to determine if proximal injury was associated with increased complication rates.

A comparison between the two groups were made using univariate statistical analysis. *P*-value < 0.05 was considered statistically significant. The project received ethics approval from the Alfred Health ethics committee prior to commencement.

## Results

We analyzed all 141 patients who underwent lower limb flap reconstruction as a result of trauma at the Alfred between 2010 and 2017 inclusive. Overall, 50 patients were identified as being in the proximal trauma group and 91 patients in the control group with no proximal trauma (Table 1).

**Table 1:** Baseline demographics for proximal trauma group and control group.

**Baseline demographics**	**Proximal trauma**	**Control**	***p*-value**
	***n* = 50**	***n* = 91**	
**Age**			
Mean (Range)	44.2 (16–80)	45.8 (21–82)	–
Smoker	12 (24.0%)	12 (13.2%)	0.10
Diabetes	4 (8.0%)	8 (8.8%)	1.0
PVD	1 (2.0%)	3 (3.3%)	1.0
**Flap**			
Muscle	13 (26.0%)	18 (19.8%)	0.39
Fasciocutaneous	37 (74.0%)	73 (80.2%)	

Fasciocutaneous flaps were more frequently used in both our proximal trauma and control groups compared to muscle or myocutaneous flaps (Table 1). Fasciocutaneous flaps were utilized in 74.0% proximal trauma and 80.2% control group patients with muscle/myocutaneous flaps used in 26.0% of proximal trauma and 19.8% of control group patients.

All trauma patients were routinely started on prophylactic dose of Enoxaparin daily on admission except one patient. This patient had suffered intra-cranial and liver hemorrhages therefore was not started on prophylactic anticoagulation until deemed safe. After the free flap reconstruction, all patients either remained on prophylactic enoxaparin or were changed to prophylactic Heparin 5,000 units BD or TDS depending on surgeon preference.

Three patients in the control and five patients in the trauma groups were changed to therapeutic anticoagulation (heparin infusion or therapeutic enoxaparin) prior to their free flap due to DVT and/or PE diagnosed during their work-up. Therapeutic anticoagulation was continued for these patients post-operatively.

Angiogram or CTA was obtained for 130 of the 141 patients included in this study. The 11 patients who did not have pre-operative arteriography were patients who had injuries at the level of the ankle/foot with clinically perfused feet. The results of the arteriography are in Table 2. Phlebography was not routinely obtained.

**Table 2:** Pre-operative arterial patency of lower limb.

**Angiogram/CTA findings**	**Proximal trauma (47 of 50 patients scanned)**	**Control (83 of 91 patients scanned)**
Three vessel run off	35	71
Two vessel run off	9	11
One vessel run off	3	1

Majority of our flaps underwent end-to-end arterial anastomosis to either posterior tibial (PTA) or anterior tibial (ATA) vessels (134 of 141 flaps). There were four flaps with end-to-side arterial anastomoses and three flaps that were anastomosed as arterial flow through flaps. Choice of recipient arteries are outlined in Table 3.

**Table 3:** Choice of recipient artery for free flap.

**Recipient artery**	**Proximal trauma (*n* = 50)**	**Control (*n* = 91)**
Posterior Tibial Artery (PTA)	22 (44.0%)	48 (52.7%)
Anterior Tibial Artery (ATA)	27 (54.0%)	37 (40.7%)
Dorsalis Pedis (DP)	0 (0.0%)	3 (3.3%)
Descending Genicular Artery (DGA)	1 (2.0%)	1 (1.1%)
Superficial Femoral Artery (SFA)	0 (0.0%)	1 (1.1%)
Popliteal Artery (PA)	0 (0.0%)	1 (1.1%)

Couplers were introduced to our unit during the course of the study. Couplers were used in 41 of the control group (45.1%) and 26 patients of the trauma group (52.0%). There was no statistically significant correlation between the introduction of couplers and the rate of venous anastomosis related complications. The veins used in microsurgery were based on surgeon preference and availability. Generally, patients had venous anastomoses to at least one vena comitans of the utilized donor artery, with or without using either a second vena comitans or a cutaneous vein. A summary of the venous recipients is shown on Table 4.

**Table 4:** Choice of recipient vein/s for free flap.

**Recipient venous anatomosis system**	**Proximal trauma (*n* = 50)**	**Control (*n* = 91)**
Vena comitans - single anastomosis	9 (18.0%)	15 (16.5%)
Vena comitans – two or three anastomoses	26 (52.0%)	50 (54.9%)
Vena comitans (one or two anastomoses) + Superficial vein (one anastomosis)	15 (30.0%)	23 (25.3%)
Superficial vein only (single anastomosis)	0 (0.0%)	3 (3.3%)

In majority of our patients, the donor choice was the contralateral leg or a distant site (upper limb or trunk) over the ipsilateral thigh (Table 5).

**Table 5:** Free flap donor site preference.

**Donor site preference**	**Proximal trauma (*n* = 50)**	**Control (*n* = 91)**
Distant	16 (32.0%)	29 (31.9%)
Contralateral lower limb	30 (60.0%)	57 (62.6%)
Ipsilateral lower limb	4 (8.0%)	5 (5.5%)

Complication rates for each group are outlined in Table 6. The most common reason for unplanned return to theater in the proximal trauma group was to explore the anastomosis which occurred in 18.0%. All of these patients had venous outflow compromise which was managed by revising the venous anastomosis either directly or utilizing vein grafts. In the control group, the return to theater rate for exploration of anastomoses was 4.4%. Venous thrombosis requiring revision was present in only half of these re-explored control cases. Overall, therefore, the study group anastomotic thrombosis rate was 18.0% but the control group thrombosis rate was 2.2%. Both groups had similar rates of return to theater for hematoma (proximal trauma 6.0% vs. control 6.6%). Total unplanned return to theater rate for all causes in the proximal trauma patients was higher at 28.0% compared to the control group 20.8% but this did not reach statistical significance. The overall flap loss rate in the proximal trauma group was ultimately 4.0%, compared to the control group at 2.2% (*p* = 0.62). All patients with a failed flap went onto have a successful reconstruction with a subsequent flap in the acute admission and there were no amputations. There were no cases of arterial insufficiency causing a return to theater in either the study or the control groups.

**Table 6:** Complication rates between proximal trauma group and control group.

**Complications**	**Proximal trauma (*n* = 50)**	**Control (*n* = 91)**	***p*-value**
Return to theater (all causes)	14 (28.0%)	19 (20.8%)	0.34
Exploration of anastomosis	9 (18.0%)	4 (4.4%)	0.013^*^
Confirmed thrombosis rate	9 (18.0%)	2 (2.2%)	0.002^*^
Partial necrosis	6 (12.0%)	4 (4.4%)	0.17
Hematoma/swelling only	3 (6.0%)	6 (6.6%)	1.00
Infection (requiring return to theater)	0 (0.0%)	2 (2.2%)	0.54
Complete flap loss	2 (4.0%)	2 (2.2%)	0.62

* *Statistically significant*.

## Discussion

Overall, there was no statistically significant difference in the rate of post-operative haematoma, swelling or infection between the proximal trauma and control groups (Table 6). To salvage the lower limb in significant trauma the initial, and most important, step is to prevent infection by early, aggressive debridement and appropriate antibiotics. This initial phase requires communication between senior plastic and orthopedic surgeons. Only two patients in the total study needed to return to theater for infection during their acute admission. Both of these patients were in the control group.

The most important finding in this study was the statically higher rate of microvascular anastomosis thrombosis in the study group (18.0%) compared to the control group (2.2%). This equates to a just over eight times higher rate of microvascular thrombosis in the proximal trauma group compared to the control. All of these patients had a venous thrombosis compromising flap perfusion. Where possible, the area of thrombosis was resected and the venous anastomosis was revised directly. In the cases where this was either not possible or deemed too high risk, a reversed vein graft was used to anastomose the flap vein to appropriately located and patent recipient vein. After any anastomotic revision, patients were started on therapeutic heparin post-operatively, unless there was a contraindication.

The higher incidence of venous thrombosis in the proximal trauma group may be related to the nature of the trauma the limb suffered. Having a multi-level injury implies that the limb has undergone a more substantial direct force with shear and crush forces applied across a larger area compared to the control group patients. In this type of injury, veins are particularly vulnerable to endothelial damage, which may not always be evident at the time of the free flap reconstruction. When these veins with endothelial injury are used in the micro-anastomosis of the free flap, their physiology may be further stressed, gradually leading to venous thrombosis. Another possibility is that those patients with proximal trauma may have had a greater incidence of occult deep venous thromboses, which contributed to increased stasis within the draining venous system and increased the risk of anastomotic thrombosis.

We had two complete free flap losses in each group. All four flap losses were following venous thrombosis. One of these four patients, also developed an infection within the flap. All four patients went onto having a successful subsequent reconstruction—one with a loco-regional flap, three with a second free flap. There were no amputations in the acute admission.

We did have minor partial necrosis in six patients from the proximal trauma group (12%) and four patients from the control group (4.4%). For the six patients in the proximal trauma group, four had minor area of flap necrosis following venous congestion and one following evacuation of a haematoma. All five of these patients underwent a minor debridement and SSG to the area of necrosis. The sixth patient from the proximal trauma group underwent debridement and loco-regional flap coverage for tip necrosis of a free flap. For the control group, on the other hand, two of the four partial necrosis cases occurred following venous congestion. The other two were flap tip necrosis. All four patients in the control group had minor partial flap necrosis which were debrided and covered with SSG.

Our unit aims to complete skeletal and soft tissue reconstruction within 1 week of injury. This, however, is not possible in those patients who have suffered other injuries that need to be addressed prior to free flap reconstruction. Godina et al. have recommended flap coverage within 3 days of injury as being associated with less infection and less flap failure (9). Subsequent studies have looked at different timeframes for early coverage. Fischer et al. defined early coverage as within 10 days alongside of another subgroup who received coverage between 11 days and 6 weeks and a third subgroup that received coverage after 6 weeks (22). The early coverage group had lower incidence of infection and shorter hospital stay. The BAPRAS/BOA guidelines on “Standard of Management of Open Fractures of the Lower Limb” recommend coverage within 7 days prior to vessels becoming friable or fibrosed (29). Numerous studies support early coverage, although the definition of early may vary from within 24 h to 15 days (23–27).

There is evidence to support the use both of fasciocutaneous and myocutaneous/muscle flap compositions and often the decision is based upon surgeon preference. Muscle flaps may provide an advantage in obliterating dead-space to prevent haematoma or seroma. There have been early studies to suggest muscle flaps may provide a higher resistance to infection and provide better vascularity which has resulted in their recommendation by some authors (30, 31). Small and Mollan (23) have reviewed 168 open tibial fractures over 15 years and have favored muscle coverage based on experimental evidence in animal models (32–35) and their own complication rates which were reported as highest in the fasciocutaneous flaps. More recent research has however refuted this premise (36–38). Paro et al. have published a retrospective study comparing their outcomes for muscle vs. fasciocutaneous free flaps (36). In a total of 86 free muscle flaps and 35 free fasciocutaneous flaps over 10 years, there was no statistically significant difference in major or minor acute complications. Muscle and fasciocutaneous flaps were comparable in outcomes in a multi-center retrospective review of 518 patients by Cho et al. (37). Cherubino et al. (38) also found no convincing evidence to support muscle or fasciocutaneous flaps in lower limb trauma in their large-scale systematic review of lower limb trauma reconstruction.

## Conclusion

This study shows a significantly greater microvascular thrombosis rate in patients with pelvic or ipsilateral limb trauma proximal to the site of a lower extremity free flap. Despite this eight-fold increase in venous thrombosis rate, the ultimate overall primary flap survival rate in this more injured study group was still 96%. We have changed our practice in light of this study, and recommend:

1. Ultrasound studies looking for limb deep vein thrombosis prior to free flap reconstruction in proximally injured patients.
2. Consideration for increased anticoagulation therapy where safe in proximally injured patients.
3. Selection whenever possible of clinically easily monitored free flaps which are either fasciocutaneous or myocutaneous for early detection of thromboses, should they occur.

We believe with appropriate judgement and case selection, that severe coexisting proximal trauma predicts a higher venous microvascular complication rate but is not a contraindication to limb salvage.

## Data Availability Statement

The raw data supporting the conclusions of this article will be made available by the authors, without undue reservation.

## Ethics Statement

The studies involving human participants were reviewed and approved by The Alfred Ethics Committee. Written informed consent for participation was not required for this study in accordance with the national legislation and the institutional requirements.

## Author Contributions

SF and FB-R conceived this study. NY collected and analyzed the data for this study. All authors contributed to the preparation of this manuscript and approved its final submission.

## Conflict of Interest

The authors declare that the research was conducted in the absence of any commercial or financial relationships that could be construed as a potential conflict of interest.
